# Advanced practice role delineation within Hong Kong: A cross‐sectional study

**DOI:** 10.1111/nhs.12964

**Published:** 2022-07-08

**Authors:** Krista Jokiniemi, Sek Ying Chair, Frances Kam Yuet Wong, Denise Bryant‐Lukosius

**Affiliations:** ^1^ Department of Nursing Science, Faculty of Health Sciences University of Eastern Finland Kuopio Finland; ^2^ The Nethersole School of Nursing, Faculty of Medicine The Chinese University of Hong Kong Hong Kong SAR China; ^3^ The Hong Kong Academy of Nursing Lai Chi Kok Hong Kong SAR China; ^4^ Faculty of Health & Social Sciences, Chair Professor of Advanced Nursing Practice, School of Nursing The Hong Kong Polytechnic University Hong Kong SAR China; ^5^ School of Nursing McMaster University Hamilton Ontario Canada

**Keywords:** advanced practice nursing, consultants, cross‐sectional studies, Clinical leadership, Nurse's role, Nursing leadership

## Abstract

A career ladder for nurses, including several levels of nursing practice and specific roles for advanced practice nurses, was introduced in Hong Kong around the start of the 21st century. To date no studies have distinguished the practices of advanced practice nurses in Hong Kong. This cross‐sectional study, conducted between November 2020 and March 2021, aims to identify and differentiate the practice patterns of advanced practice nurses by utilizing the Advanced Practice Role Delineation tool. A total of 191 responses were obtained. Three roles were identified: nurse consultant, advanced practice nurse, and advanced practice nurse in management. Among the five advanced practice nursing domains, nurses were most frequently involved in Education and in Direct Comprehensive Care activities, while least active in Research and in Publication and Professional Leadership. Identifying activities in various nursing roles helps to differentiate their responsibilities and provides new insights for role utilization and support. Although the role characteristics are shaped by country contexts, research evidence on practice patterns may be used to support international discussion and efforts to promote role clarity and effective role introduction and optimization.

**Key points**

- Several countries, including Hong Kong, have deployed specific roles for advanced practice nurses. Identification of different advanced practice nursing role activities helps to differentiate responsibilities and provides new insights for role utilization. It is necessary to continue to distinguish and specify these roles to ensure consistent implementation and integration of these roles within the clinical nursing career ladder.
- Study results build the international knowledge base on the differentiation of advanced practice nursing roles to promote understanding of various roles and improve role clarity. The research evidence on advanced practice nursing practice patterns may be used by nurse managers to support effective role introduction and role optimization, service planners to match existing nursing position titles to the needs of the services, and by educators to design postgraduate courses responsive to the clinical career ladder of nurses.

## 1 INTRODUCTION

Increasingly, countries are challenged to provide access to high quality care and are seeking ways to optimize provider expertise to meet demands while containing healthcare costs (International Council of Nurses [ICN], 2020; World Health Organization [WHO], 2020). There is a need for clearer understanding of the practice patterns of nurses in advanced roles to assist healthcare managers in clarifying and making effective decisions about how to best utilize and integrate these various roles within organizations. In this article, we will describe the advanced practice nursing workforce within Hong Kong and outline study results identifying and differentiating practice activities among recognized advanced practice nursing roles.

## 2 BACKGROUND

Specialist and advanced practice beyond generalist nursing have become internationally recognized nursing phenomena during the last few decades. However, a great variation among different levels of nursing roles exists between and within countries, as countries are in different stages of advanced practice nursing role development (ICN, 2020; Maier et al., 2017). Although all nurses contribute to the healthcare system through their different roles, those in advanced practice nursing roles build on registered nurse (RN) practice by integrating higher level knowledge, skills, and experience across several clinical and non‐clinical role domains with the ultimate aim to meet population health needs and improve health and health system outcomes (Bryant‐Lukosius & Wong, 2019; ICN, 2020). One of the most significant barriers to effective advanced practice nursing role implementation and utilization is the lack of role clarity (Mohr & Coke, 2018). The ICN defines an advanced practice nurse (APN) as “a generalist or specialised nurse who has acquired, through additional graduate education (minimum of a master's degree), the expert knowledge base, complex decision‐making skills and clinical competencies for Advanced Nursing Practice, the characteristics of which are shaped by the context in which they are credentialed to practice” (ICN, 2020, pp. 6).

There are about 46 000 RNs eligible to practice in the Hong Kong (Nursing Council of Hong Kong, 2020b). A career ladder for nurses was introduced around the start of the 21st century to respond to the challenges of complex client care and the advancement of healthcare (Wong & Wong, 2020). The first advanced practice nursing role was piloted in 2003 with the majority of positions occurring in inpatient settings (Parker & Hill, 2017). Currently, there are approximately 5000 APNs in Hong Kong (Hospital Authority, 2021a). While APNs do not have licensure beyond that of RNs, the career ladder of the RN, specialist nurse (SN), APN, and nurse consultant (NC) are clearly set out (Parker & Hill, 2017). The professional career ladder is a three‐tier pathway. Tier 1 applies for RNs and SNs, tier 2 applies to the APNs/ward managers, and tier 3 to NCs (Chan, 2014; Parker & Hill, 2017). Two streams of career progression are provided through the system: management or clinical (Parker & Hill, 2017).

The Nursing Council of Hong Kong (2020b) states that RNs should be eligible to apply for recognition as an APN if they have:

(a) obtained a post‐RN registration *Clinical Master in Nursing/Health Science in the related specialty; or (b) … Master degree in health related stream* AND completed the Post‐registration Certificate Course (“PRCC”)/Hospital Authority's Specialty Nurse Recognition Scheme Certificate/recognised in‐service training for at least 80 hours; or (c) is a Fellow of the Hong Kong Academy of Nursing or equivalent; AND (d) possesses six years of full time post‐registration nursing experience immediately prior to his/her application in which at least the most recent 4 years must be serving in the related specialty area. (Section “Who can apply?” on the webpage “Voluntary Scheme on Advanced and Specialised Nursing Practice”; emphasis added)

Although a Master's degree is a minimum requirement for APN recognition, some nurses may not meet this standard due to lack of formal credentialing processes and the recent development of educational standards for the APN in Hong Kong (Wong & Wong, 2020).

The NC role was introduced in Hong Kong in 2009 to model the Clinical Nurse Specialist (CNS) role in the United States (Wong et al., 2017). NCs are required to have a master's education, specialty training, and eight years of work experience (Lee et al., 2013), and are seen to practice at the highest level of a clinical nursing career (Gardner et al., 2017; Lee et al., 2013; Wong et al., 2017). Evaluations of the NC in Hong Kong demonstrate their exemplary work and positive impact on patient, nursing profession, and organization outcomes (Wong et al., 2017). NCs are involved in developing and providing complex patient care and leading quality improvement initiatives. Five main categories of roles and responsibilities of NCs have been recognized: expert practitioner, service developer and planner, quality assurer, educator, and researcher (Chan, 2014). The number of NCs in Hong Kong is around 160 and recently the Hospital Authority has announced strategies to retain staff by increasing the number of NCs and associate NCs to 200 and 442, respectively. The introduction of the associate NC rank, as a step prior to NC, helps to facilitate continuous development of nursing talents and promote nursing service standards (Hospital Authority, 2021b).

The APN in management (APN‐M) is one of 16 specialties in advanced nursing practice in Hong Kong. This position performs a specialist role in a clinical specialty, providing management in advanced nursing care and acting as a resource person to patients, relatives, staff, and the public, as appropriate. APN‐Ms also engage in research activities and promote evidence‐based practice for the betterment of service (Hospital Authority, n.d.) APN‐Ms and NCs share common competencies developed by the Hong Kong Academy of Nursing in 2018 (Wong & Wong, 2020). These competencies align with international standards for advanced practice nursing and promote role clarity and integration within Hong Kong (Wong et al., 2017). The role activities of APN‐Ms cover the scope of five core competence areas comprising (1) professional, legal, and ethical nursing practice; (2) health promotion and health education; (3) management and leadership; (4) evidence‐based practice and research; and (5) personal and professional development. Within APN‐M roles, there can be differences in emphasis on role responsibilities related to clinical practice, management, education and mentorship, and research (Nursing Council of Hong Kong, 2020a).

With the development of specialist and advanced practice nursing roles, it is necessary to distinguish and specify these roles in the professional promotion ladders (Hill et al., 2017). This comparative descriptive study aimed to identify and distinguish the practice patterns of APNs within Hong Kong by utilizing the Advanced Practice Role Delineation (APRD) tool (Gardner et al., 2013). The APRD tool has been used internationally to distinguish nursing and advanced practice nursing roles (Jokiniemi et al., 2022) in several countries, for example in the United States, Australia, Finland, Canada, Spain, New Zealand, and Singapore (Carryer et al., 2018; Gardner et al., 2013; Jokiniemi et al., 2018; Jokiniemi, Heikkilä, et al., 2021a; Mick & Ackerman, 2000; Sevilla Guerra, Miranda Salmerón, & Zabalegui, 2018a; Woo et al., 2019). The results of this study may be used to support evidence‐informed decision‐making and role optimization through improved understanding about the unique and complementary nature of these roles. This research is aligned with the strategic priorities of the WHO (2015) to effectively introduce advanced roles for nurses within healthcare systems.

## 3 METHODS

### 3.1 Research design

A cross‐sectional study was conducted using an online self‐report questionnaire. To report the study, the Strengthening the Reporting of Observation studies in Epidemiology (STROBE) checklist was utilized (File S1 in the Supporting Information).

### 3.2 Data collection and participants

The data collection was conducted in Hong Kong between November 2020 and March 2021. To be eligible for this study, participants had to be (a) working in a clinical role, and (b) certified in a specialized area of practice with a master's or doctorate degree. To reach a wide range of APNs the invitation to participate in the study, including an information letter and link to the survey, was sent to the members of the Hong Kong Academy of Nursing (*n* = 2546) and advanced practice nursing postgraduate students (*n* = 313) through a Hong Kong Academy of Nursing contact person who is also an author of this paper. A total of four remainder e‐mails were sent out through the contact person.

### 3.3 Measurements

The questionnaire had three sections: (1) sociodemographic data on professional (i.e., role title, practice experience, certification, area of practice) and educational (specialty education, highest level of nursing education) qualifications; (2) the 41‐item APRD tool identifying practice activities in the five domains of Direct Comprehensive Care, Support of Systems, Education, Research, and Professional Leadership; and (3) perceptions on time spent overall in each of the domains. In Sections 2 and 3, a 5‐point Likert‐type scale (0 = never, 1 = rarely, 2 = sometimes, 3 = often, and 4 = always) was used to measure the use of nursing activities or overall time spent in domains of practice during a typical month (Gardner et al., 2013).

The content of the APRD tool has been studied in Australia (Chang et al., 2010), Finland (Jokiniemi, Ackerman, et al., 2021b), and Spain (Sevilla Guerra et al., 2018a, b). The construct of the tool has been examined by exploratory factor analysis (Chang et al., 2012) and confirmatory factor analysis (Jokiniemi et al., 2022; Sevilla Guerra, Risco Vilarasau, et al., 2018b). Although some changes have been suggested in previous studies, the original tool was used in this study to allow for comparison across studies using the APRD tool. The Cronbach's alpha scores for the APRD tool have been found to be between 0.90 (Jokiniemi, Heikkilä, et al., 2021a) and 0.94 (Chang et al., 2012), indicating strong internal consistency for the scale. In 2019, a pilot test of the instrument was conducted in Hong Kong (*n* = 12). Only minor amendments to the questionnaire were required to improve understandability of the language for local readers

### 3.4 Statistical analysis

The Statistical Package for the Social Sciences (SPSS, version 27.0, 2017) was used to analyze the data. There were no missing data, so therefore raw data could be used to analyze the results. To describe the characteristics of the sample, descriptive statistics were used. Categorical variables are presented as counts and percentages and continuous variables are presented as means. For Section 2 of the questionnaire, the sum of each activity in the APRD tool and the mean scores for each subscale were calculated to illustrate their use. For the purpose of analysis, role titles of the participants were organized into the three categories of NC, APN, and APN‐M. Based on role category, role differences related to activities in the five domains were compared using a one‐way analysis of variance (ANOVA) with a *p*‐value of less than 0.05 to establish significance. ANOVA between‐role effects taking into account respondents’ gender, age, education, experience in current position, and type of hospital were explored. Tukey's HSD post hoc test was used to explore the statistically significant differences between role categories. Age was organized into three groups (from lowest to 40 years; 41 to 50 years; and from 51 years and over), and years of experience in current position was categorized into three groups (from 0 to 2 years [novice/beginner]; 2 to 4 years [competent/proficient]; and 5 years and above [approaching expert/expert]). Prior to analysis the assumptions of ANOVA were tested (homoskedasticity, normality). Data were not normally distributed; however, this was not a critical assumption in multivariable analysis. Finally, for Section 3 of the questionnaire, we calculated an overall mean score for time spent in each domain of practice.

### 3.5 Ethical considerations

The study protocol was approved by the University of Eastern Finland Committee on Research Ethics (statement number 22/2018). The Hong Kong Academy of Nurses granted permission to conduct the study with its members. Data were handled in strict confidence and disposed of in accordance with the policy guidelines of the Hong Kong Academy of Nursing. The prospective participants were informed of the study in writing. At the beginning of the survey, participants were asked to indicate their consent to be involved in the study by checking “Yes” to a consent statement. Participants could leave the study at any point.

## 4 RESULTS

### 4.1 Participant characteristics

A total of 2850 questionnaires were distributed, of which 191 were completed for a total response rate of 7%. The majority (81%) of respondents were female with an average age of 47 years. They reported working in different roles involving 13 position titles such as NC, midwife consultant, deputy chief nurse, APN, and ward manager. The position titles were re‐grouped into the three role categories of NC (*n* = 34), APNs (*n* = 106), and APN‐M (*n* = 51) aligning the two streams of career progression (management or clinical) and accommodating the second (APNs/nurse managers) and third (NCs) tiers of the career ladder. The majority of nurses (92.2%) had a master's or doctoral degree and worked in an acute hospital (81%). Respondents had worked as a RN for an average of 25.6 years and had been in their current APN position for an average of 6.2 years. Nurse consultants were most educated and experienced out of the three groups (Table 1). Over 80% of the respondents provided direct patient care with 98 percent working in full‐time positions. Several specialties were recognized with the largest three being critical care (*n* = 10), cardiac care (*n* = 9), and community and public health (*n* = 7).

**TABLE 1:** Demographic characteristics of the participants.

Title (*n* = 191)	All (*n* = 191)		NC (*n* = 34)		APN (*n* = 106)		APN‐M (*n* = 51)	
Characteristic	*n*	%	*n*	%	*n*	%	*n*	%
Female	154	80.6	25	73.5	85	80.2	44	86.3
Hospital type (*n* = 191)								
Acute	154	80.6	30	88.2	89	84.0	35	68.6
Rehabilitation	19	9.9	0	0	10	9.4	9	17.6
Day care	6	3.1	3	8.8	1	0.9	2	3.9
N/A	11	5.8	1	2.9	5	9.8	5	4.7
Highest level of education (*n* = 1491)								
Undergraduate degree	14	7.3	0	0	8	7.5	6	11.8
Master's	172	89.6	29	85.3	98	92.5	45	88.2
Doctoral	5	2.6	5	14.7	0	0	0	0

	Mean	SD	Mean	SD	Mean	SD	Mean	SD
Age	47.01	7.61	52.53	4.64	44.99	6.22	47.53	9.75
Years in nursing	25.67	7.93	30.65	4.98	23.30	6.82	27.56	9.68
Years in current position	6.24	4.90	4.49	3.48	7.65	5.08	4.50	4.43

Abbreviations: APN, advanced practice nurse; APN‐M, advanced practice nurse in management; NC, nurse consultant.

### 4.2 Comparison of advanced practice nursing activities

**FIGURE 1:**
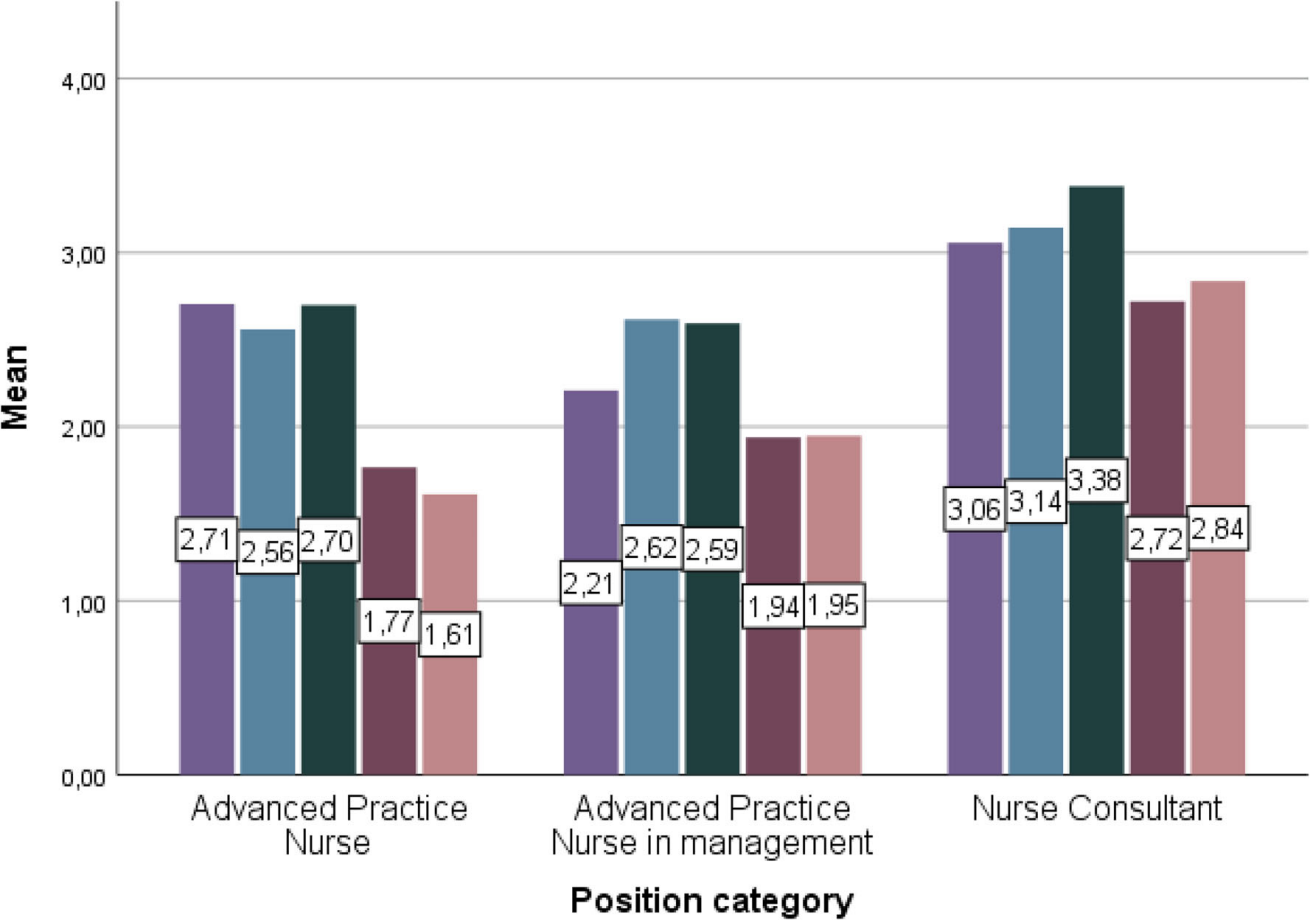
Mean scores for advanced practice nursing domain activity according to role category. 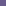, Direct comprehensive care; 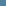, Support of systems; 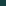, Education; 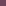, Research; 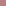, Publication and professional leadership.

The Cronbach's alpha coefficient for the APRD scale was 0.966 in this study, indicating strong internal consistency. The subscale Cronbach's alpha scores ranged from 0.958 (Direct Comprehensive Care) as the highest to 0.889 (Education) as lowest. Based on the total mean scores for each domain, the nurses were most active in Education (total mean 2.80 out of 4), Direct Comprehensive Care (total mean 2.63), and Support of Systems (total mean 2.68). The two least utilized domains of practice were Research (total mean 1.99) and Publication and Professional Leadership (total mean 1.93). The ANOVA between‐role effects showed statistically significant differences in all of the observed five activities (*p* < 0.05) when gender, age, education, experience in current position, and hospital type were taken into account. Nurse consultants were significantly more involved in all of the domains compared to APNs and APN‐Ms (*p* < 0.05 for Direct Comprehensive Care, and *p* < 0.001 for the other four domains; Figure 1).

#### 4.2.1 Direct comprehensive care

All APNs had high scores in Direct Comprehensive Care activity with the three role category means ranging from 2.21 for APN‐Ms to 3.06 for NCs (Figure 1). Nurse consultants were most frequently involved in all activities within this domain except Item 1.12, “Facilitate the process of ethical decision‐making in patient care,” where APNs had a slightly higher score. In keeping with their position title, the NCs’ highest activity score was on Item 1.11, “Serve as a consultant in improving patient care and nursing/midwifery practice based on expertise in area of specialization.” The APN‐Ms had the lowest activity scores within the Direct Comprehensive Care domain. Based on Tukey post hoc analysis, statistically significant differences between role categories were found in all 15 items. In 13 of the 15 items, APNs and NCs did not differ significantly from each other (Table 2). The ANOVA between‐role effect for Direct Comprehensive Care showed statistically significant differences between role categories (*p* < 0.05) when gender, age, education, experience in current position, and hospital type were taken into account. Nineteen percent of the variability in the Direct Comprehensive Care activity could be accounted for by the role category of the respondent.

**TABLE 2:** Mean scores and statistically significant differences (ANOVA) between groups

Activity	Role						ANOVA
	APN		APN in management		Nurse consultant		BG
	Mean	SD	Mean	SD	Mean	SD	*p*<
**Direct comprehensive care**							
1.1 Conduct and document patient history and physical examination	2.62	0.93	2.04	1.26	**3.06**	0.98	***
1.2 Assess psychosocial, cultural and religious factors affecting patient needs	2.36	0.93	1.84	1.19	**2.76**	1.05	***
1.3	2.14	1.06	1.92	1.18	**2.65**	1.13	*
1.4 Identify and initiate required diagnostic tests and procedures	2.25	0.96	1.88	1.18	**2.59**	1.16	*
1.5 Gather and interpret assessment data to formulate plan of care	2.71	0.87	2.16	1.12	**3.15**	0.78	***
1.6 Perform specialty‐specific care and procedures	3.05	0.87	2.27	1.22	**3.24**	1.02	***
1.7 Assess patient/family response to therapy and modify plan of care based on response	2.76	0.91	2.10	1.24	**2.97**	1.00	***
1.8 Communicate plan of care and response to patient/family	2.89	0.93	2.12	1.24	**3.15**	0.93	***
1.9 Provide appropriate education (counseling) to patient & family	2.84	0.93	2.12	1.24	**3.24**	0.96	***
1.10 Document appropriately on patient record	2.95	0.96	2.25	1.25	**3.15**	0.96	***
1.11 Serve as a consultant in improving patient care and nursing/midwifery practice based on expertise in area of specialization	2.73	0.97	2.37	1.08	**3.59**	0.74	***
1.12 Facilitate the process of ethical decision making in patient care	**2.75**	0.85	2.29	1.10	2.74	0.83	*
1.13 Coordinate interdisciplinary plan for care of patients	2.91	0.82	2.49	1.12	**3.24**	0.74	*
1.14 Collaborate with other services to optimize patient's health status	2.84	0.77	2.67	1.14	**3.24**	0.78	*
1.15 Facilitate efficient movement of patient through healthcare system	2.79	0.83	2.59	1.10	**3.12**	0.81	*
Support of systems							
2.1 Consult with others regarding conduct of projects or presentations	2.36	0.91	2.35	0.91	**2.85**	0.78	*
2.2 Contribute to, consult or collaborate with other healthcare personnel on recruitment and retention activities	2.17	1.14	**2.51**	0.95	2.41	1.08	
2.3 Participate in strategic planning for the service, department or hospital	2.20	0.94	2.63	1.02	**2.97**	0.94	***
2.4 Provide direction for and participation in unit/service quality improvement programs	2.50	0.88	2.71	1.01	**3.41**	0.82	***
2.5 Actively participate in the assessment, development, implementation, and evaluation of quality‐improvement programs in collaboration with nursing/midwifery leadership	2.53	0.95	2.65	1.04	**3.35**	0.77	***
2.6 Serve as a mentor	3.26	0.72	2.69	0.95	**3.56**	0.66	***
2.7 Advocate the role of the nurse/midwife	3.01	0.90	2.78	0.78	**3.44**	0.71	*
2.8 Serve as a spokesperson for nursing/midwifery and the health facility when interacting with other professionals, patients, families, and the public	2.44	1.10	2.63	0.89	**3.15**	0.74	*
Education							
3.1 Evaluate education programs and recommend revision as needed	2.35	1.07	2.63	0.87	**3.29**	0.72	***
3.2 Serve as educator and clinical preceptor for nursing/midwifery and/or medical students, staff, and/or others	2.92	0.82	2.61	0.90	**3.56**	0.61	***
3.3 Identify learning needs of various populations and contribute to the development of educational programs/resources	2.42	1.11	2.61	1.04	**3.32**	0.59	***
3.4 Serve as informal educator to staff while providing direct care activities	3.07	0.77	2.63	1.00	**3.50**	0.75	***
3.5 Facilitate professional development of nursing/midwifery staff through education	2.55	0.97	2.73	0.96	**3.44**	0.71	***
3.6 Provide appropriate patient and family education	2.91	0.83	2.37	1.10	**3.18**	0.97	***
Research							
4.1 Conduct clinical research	1.26	0.96	1.39	1.04	**2.47**	0.79	***
4.2 Participate in audits to monitor and improve quality of patients care practices	2.33	0.99	2.47	1.14	**3.09**	0.79	*
4.3 Contributes to identification of potential funding sources for the development and implementation of clinical projects/programs	1.50	1.23	1.65	1.18	**2.15**	0.96	*
4.4 Uses research evidence to guide practice and policy changes	1.99	1.03	2.18	0.97	**3.21**	0.73	***
4.5 Identify the clinical data that needs to be collated and available in information systems for nursing and midwifery research and quality assurance projects	1.87	1.10	1.96	1.18	**2.94**	0.85	***
4.6 Collaborate with Information Specialists in the design of information systems for research and quality assurance projects in nursing and midwifery	1.65	1.16	1.98	1.24	**2.47**	0.99	*
Publications and professional leadership							
5.1 Disseminate nursing/midwifery knowledge through presentation or publication at local, regional, national and international levels	1.72	1.16	2.02	1.18	**2.94**	0.74	***
5.2 Serve as a resource or committee member in professional organizations	1.75	1.16	2.31	1.14	**3.18**	0.72	***
5.3 Serve as a consultant to individuals and groups within the professional/lay communities and other hospitals/institutions	1.67	1.23	2.14	1.11	**3.18**	0.76	***
5.4 Represent nursing/midwifery in institutional/community forums focused on the educational needs of various populations	1.53	1.26	1.76	1.26	**2.68**	0.84	***
5.5 Represent a professional nursing/midwifery image at institutional and community forums	1.49	1.32	1.73	1.25	**2.76**	0.92	***
5.6 Collaborate with other healthcare professionals to provide leadership in shaping public policy on healthcare	1.52	1.27	1.73	1.23	**2.29**	0.84	*

*Note*: Highest score for each item is in boldface. Abbreviations: ANOVA, analysis of variance; APN, advanced practice nurse; BG, between groups; *p < 0.05. ***p < 0.001.

#### 4.2.2 Support of systems

Compared to the other domains, there was less group variability in time spent on Support of Systems activities across the three role categories, ranging from an APN mean of 2.56 to a NC mean of 3.14. NCs were most active in the Support of Systems for seven out of eight activities (Figure 1, Table 2). APN‐Ms had a slightly higher activity score on Item 2.2, “Contribute to, consult or collaborate with other healthcare personnel on recruitment and retention activities.” In general, APNs (mean 2.56) and APN‐Ms (mean 2.62) were also highly active in the Support of Systems domain, with APN‐Ms scoring higher than APNs in five out of eight items. Based on Tukey post hoc analysis, statistically significant differences between role categories were found in seven of eight items. APNs and APN‐Ms did not differ significantly in six of the eight Support of Systems items. The ANOVA between‐role effect for Support of Systems showed statistically significant differences between the role categories (*p* < 0.001) when gender, age, education, experience in current position, and hospital type were taken into account. Seven percent of the variability in the Support of Systems activity could be accounted for by the role category of the respondent.

#### 4.2.3 Education

Again, NCs were most active in the Education domain (mean 3.38) with the highest involvement in all activities (Table 2). Based on Tukey post hoc analysis, statistically significant differences between role categories were found in all six items (Table 2). Again, APNs and APN‐Ms did not differ significantly in four of the six items. The ANOVA between‐role effect for Education showed statistically significant differences between role categories (*p* < 0.001) when gender, age, education, experience in current position, and hospital type were taken into account. Eleven percent of the variability in the Education activity could be accounted for by the role category of the respondent.

#### 4.2.4 Research

Nurse consultants were the most active in the Research domain (mean 2.72), having the highest involvement in all activities (Table 2). While APNs and APN‐Ms were most active in Item 4.2, “Participate in audits to monitor and improve quality of patients care practices,” the highest activity score of NCs was for Item 4.4, “Uses research evidence to guide practice and policy changes” (Table 2). Based on Tukey post hoc analysis, statistically significant differences between role categories were found in all six items. APNs and APN‐Ms did not differ in any of the items. The ANOVA between‐role effect for Research showed statistically significant differences between role categories (*p* < 0.001) when gender, age, education, experience in current position, and hospital type were taken into account. Fourteen percent of the variability in the Research activity could be accounted for by the role category of the respondent.

#### 4.2.5 Publication and professional leadership

Finally, NCs were the most active in the Publication and Professional Leadership domain (mean 2.84), and had the highest involvement in all activities across the three groups (Table 2). APN‐Ms were more active in each of the Publication and Professional Leadership activities than APNs. For all three groups the highest activity sore was seen on Item 5.2, “Serve as a resource or committee member in professional organisations.” There were statistically significant differences between role categories in all six items (Table 2). APNs and APN‐Ms did not differ significantly in three of the six items. The ANOVA between‐role effect for Publication and Professional Leadership showed statistically significant differences between role categories (*p* < 0.001) when gender, age, education, experience in current position, and hospital type were taken into account. Nine percent of the variability in the Publication and Professional Leadership activity could be accounted for by the role category of the respondent.

### 4.3 Perceptions of time spent in the domains

Section 3 of the questionnaire measured the use of overall time spent in the domains of practice during a typical month. According to participant self‐evaluation, NCs spent most of their time on the Education domain (mean 3.38 on a scale of 4), followed by the Direct Comprehensive Care (mean 3.21) and Support of Systems (3.15) domains. Similarly, APN‐Ms spent most of their time in Education (mean 2.49) and least in Research (1.90). In turn, APNs perceived spending the most time on Direct Comprehensive Care (mean 2.93) and the least in Publication and Professional leadership (1.82). The self‐reported activity scores (Section 2 of the questionnaire, Figure 1) and perceptions of time spent in their respective domains of practice (Section 3 of the questionnaire) were in line with each other.

## 5 DISCUSSION

This study identifies and differentiates the practice activities of APNs in various roles within Hong Kong. Approximately half of the 5000 APNs working in Hong Kong were invited to participate in this study. Despite the low response rate, the sample reflects the proportion of various roles within the current Hong Kong advanced practice nursing workforce. For example, the distribution of respondents outlined in Table 1 is consistent with workforce data indicating fewer numbers of NCs and APN‐Ms compared to APNs (Hospital Authority, 2021a).

The respondents reported thirteen different titles which were categorized into three groups aligning with the Hong Kong three‐tier career ladder. An array of titles has been acknowledged with previous studies investigating advanced practice nursing roles (Donald et al., 2010). Internationally, to improve role clarity and understanding among advanced practice nursing stakeholders and to provide a foundation for nursing career planning and development, standardization of nursing titles within and across countries is needed (Gardner et al., 2017; ICN, 2020). Nursing career ladders may be country specific and therefore consideration needs to be given to extrapolate the discussions to the international advanced practice nursing community. For example, the APN‐M role is unique to the career ladder for APNs in Hong Kong, and thus may not be transferable to advanced practice nursing roles in other countries. Despite the management focus of this role, study results demonstrate that APN‐Ms operationalize all domains of advanced practice nursing, including clinical practice. These findings suggest that APN‐Ms do meet the international definition for advanced practice nursing within the context of the Hong Kong healthcare system. Only through discussion and discernment of various advanced practice nursing roles and titling in different countries may we begin to increase our understanding and promote greater clarity and consistency of advanced practice nursing roles internationally.

About 8% of the nurses who participated in this study had not completed an MSc as an expected requirement for advanced practice. It is possible that some of these nurses were graduate students in master's programs who were already working in an advanced practice nursing role. Although a master's degree is a minimum requirement for APNs in Hong Kong (The Nursing Council of Hong Kong, 2020b), there is no formal credentialing process in place to enforce this requirement in practice (Wong & Wong, 2020). While internationally, a master's degree education is valued within the APN community, many studies report variability in APN education, especially when credential requirements are not in place (see e.g., Gardner et al., 2016; Jokiniemi et al., 2018; Jokiniemi, Heikkilä, et al., 2021a). While research regarding advanced practice nursing education is limited, a few studies have demonstrated that completion of a master's degree is important for promoting the optimal implementation of advanced practice nursing role domains (Kilpatrick et al., 2013; Pauly et al., 2004).

Across the three role categories, APNs were highly involved in activities for each of the five domains of advanced practice. Overall, nurses were most frequently engaged in Education activities. In order to deliver high quality care, nurses are expected to continually update their knowledge and skills to maintain their competency to practice. As APNs are found to focus on educating, precepting, mentoring, and facilitating the professional development of nurses and nursing students (Price & Reichert, 2017), our finding of high APN activity related to Education is not surprising. Furthermore, our finding of high APN education activity parallels the results of the most recent CNS census study in the United States, in which CNSs reported the highest amount of time (up to 32.5%) precepting nursing students. Teaching nurses/staff and patients/families was also among the top six activities for CNSs (National Association of Clinical Nurse Specialists, 2020).

The second most frequent activity, Direct Comprehensive Care, is aligned with other international studies investigating advanced practice nursing roles (Carryer et al., 2018; Gardner et al., 2016; Woo et al., 2019). This finding is also mirrored in a recent study by Chun et al. (2021), which found that APNs were most frequently involved in providing direct client services. The high level of respondents' Direct Comprehensive Care activities is reflective of the policy detailing clinical practice as the primary focus of advanced practice nursing roles within Hong Kong (Nursing Council of Hong Kong, 2020a).

Compared to APNs and APN‐Ms, NCs were the most active in all domains of advanced practice nursing, thus highlighting their exemplary position among advanced practitioners in Hong Kong. NC activity scores were statistically significantly higher (in 39 out of 41 items) from those of APNs and APN‐Ms. Previous findings demonstrating NCs as the most highly educated and experienced APNs in Hong Kong (Lee et al., 2013; Wong et al., 2017) were corroborated through our findings. Across all three groups, the majority of nurses were prepared with master's level education; however, 15% of the NCs had PhD training and were older (mean 52.5 years) in comparison to the APN and APN‐M groups, and had over 30 years of nursing work experience. NCs are also reported to be at the top of the APN career ladder in other countries beyond Hong Kong (Gardner et al., 2017; Kennedy, 2012). The NC role has been modeled after the CNS role in the United States. The areas of NC impact, in relation to patients, nurses, organizations, and systems, are similar to those of CNSs in the United States. (Bryant‐Lukosius & Wong, 2019; Jokiniemi et al., 2012). We should note, in relation to the APN, that there are no roles similar to the Nurse Practitioner role in Hong Kong.

The results of this study discern the similarities and differences in practice activities across three groups of APNs and highlight the role of the NC as a clinical leader of APNs within Hong Kong. The results offer insights for delineating and clarifying the background and activities of the newly developed advanced practice nursing roles and highlight the importance of building a career ladder for nurses to support effective role development and implementation. The successful introduction of advanced practice nursing roles in Hong Kong requires APN education and competence that match population health and practice setting needs, and also systems level health policies that optimize the utilization of APN expertise and scope of practice (Wong & Wong, 2020). Identifying advanced practice nursing activities among different advanced practice nursing roles helps to differentiate their responsibilities and provides new insights for role utilization and support.

### 5.1 Study limitations and implications for future research

Typical of online surveys, the response rate of the survey was low despite the use of multiple reminders. The low response rate may be attributed to the practice demands on nurses associated with the global COVID‐19 pandemic. The sample of the study was, however, adequate for the methodology. Furthermore, the distribution outlined in Table 1 (NC = 34, APN = 106, and APN‐M = 51) seems representative of the current advanced practice nursing workforce in Hong Kong. To address the issue of possible selection bias, we were transparent with the recruitment process, participant characteristics, and our findings to allow the reader to assess the representativeness of the sample and applicability of the results.

It is recognized that self‐reported measures may affect reliability, with participants' over‐ or underestimating observed items. To mitigate this limitation the activity scores were confirmed by inquiring respondent perceptions of overall time spent in the domains. These perceptions align with the participant self‐evaluations of the overall use of time in activities. Only the advanced practice nursing roles were examined within this study. In the future, it is important to differentiate the generalist and SN activities in relation to APN activities.

## 6 CONCLUSION

APNs in in Hong Kong actively engage in all five domains of advanced practice as defined by the APRD tool. NCs were shown to be high performers among APNs with the highest level of role activity across each of the practice domains. The research evidence on the advanced practice nursing practice patterns may be used by nurse managers to support effective role introduction and role optimization, service planners to match existing nursing position titles to the needs of the services, and by educators to design postgraduate courses responsive to the clinical career ladder of nurses. Career development and progression of nurses invigorate the optimal contribution of nurses to client care and service quality. The study results add to the international literature aiming to delineate the varied advanced practice nursing roles. As career ladders and roles are country specific, consideration needs to be given on the generalizability of the results in the international advanced practice nursing community. However, it is necessary to distinguish and specify various advanced practice nursing roles to ensure the consistent implementation and integration of these roles within the clinical nursing career ladder. Identifying activities in different nursing positions will help to differentiate these roles and responsibilities and aid in the governance and clarity of these roles.

## AUTHOR CONTRIBUTIONS

Krista Jokiniemi, Sek Ying Chair, Frances Kam Yuet Wong, Denise Bryant‐Lukosius: Made substantial contributions to the conception and design, or acquisition of data, or analysis and interpretation of data. Krista Jokiniemi, Sek Ying Chair, Frances Kam Yuet Wong, Denise Bryant‐Lukosius: Involved in drafting the manuscript or revising it critically for important intellectual content. Krista Jokiniemi, Sek Ying Chair, Frances Kam Yuet Wong, Denise Bryant‐Lukosius: Gave final approval of the version to be published. Each author has participated sufficiently in the work to take public responsibility for appropriate portions of the content; Krista Jokiniemi, Sek Ying Chair, Frances Kam Yuet Wong, Denise Bryant‐Lukosius agree to be accountable for all aspects of the work in ensuring that questions related to the accuracy or integrity of any part of the work are appropriately investigated and resolved.

## FUNDING INFORMATION

The study received no specific funding.

## CONFLICT OF INTEREST

No conflict of interest has been declared by the authors.

## Supporting information


**File S1** Supporting information

## Data Availability

Author elects to not share data
